# Anti-Parallel β-Hairpin Structure in Soluble Aβ Oligomers of Aβ40-Dutch and Aβ40-Iowa

**DOI:** 10.3390/ijms22031225

**Published:** 2021-01-27

**Authors:** Ziao Fu, William E. Van Nostrand, Steven O. Smith

**Affiliations:** 1 Center for Structural Biology, Department of Biochemistry and Cell Biology, Stony Brook University, Stony Brook, NY 11794, USA; zfu@mail.rockefeller.edu; 2 George and Anne Ryan Institute for Neuroscience, University of Rhode Island, Kingston, RI 02881, USA; wvannostrand@uri.edu; 3 Department of Biomedical and Pharmaceutical Sciences, University of Rhode Island, Kingston, RI 02881, USA

**Keywords:** Alzheimer’s disease, cerebral amyloid angiopathy, amyloid-β, Aβ40-Dutch, Aβ40-Iowa

## Abstract

The amyloid-β (Aβ) peptides are associated with two prominent diseases in the brain, Alzheimer’s disease (AD) and cerebral amyloid angiopathy (CAA). Aβ42 is the dominant component of cored parenchymal plaques associated with AD, while Aβ40 is the predominant component of vascular amyloid associated with CAA. There are familial CAA mutations at positions Glu22 and Asp23 that lead to aggressive Aβ aggregation, drive vascular amyloid deposition and result in degradation of vascular membranes. In this study, we compared the transition of the monomeric Aβ40-WT peptide into soluble oligomers and fibrils with the corresponding transitions of the Aβ40-Dutch (E22Q), Aβ40-Iowa (D23N) and Aβ40-Dutch, Iowa (E22Q, D23N) mutants. FTIR measurements show that in a fashion similar to Aβ40-WT, the familial CAA mutants form transient intermediates with anti-parallel β-structure. This structure appears before the formation of cross-β-sheet fibrils as determined by thioflavin T fluorescence and circular dichroism spectroscopy and occurs when AFM images reveal the presence of soluble oligomers and protofibrils. Although the anti-parallel β-hairpin is a common intermediate on the pathway to Aβ fibrils for the four peptides studied, the rate of conversion to cross-β-sheet fibril structure differs for each.

## 1. Introduction

Sequential cleavage of the amyloid precursor protein (APP) by two proteases generates the amyloid-β (Aβ) peptides associated with Alzheimer’s disease (AD) [1]. The first cleavage between the extracellular and the transmembrane (TM) domain of APP by β-secretase generates an N-terminal soluble fragment and a membrane-anchored C-terminal fragment (CTF) [2,3]. A second protease (γ-secretase) cleaves the CTF within the TM domain to generate the Aβ peptides [4,5]. The γ-secretase cleavage site is not specific. The 40-residue Aβ40 peptide is the predominant cleavage product representing ~90% of the total secreted Aβ [6]. However, the 42-residue Aβ42 peptide represents ~5–10% of secreted Aβ. Aβ42 is considered to be the most neurotoxic form of Aβ, rapidly aggregates [7,8] and is the principal component of amyloid plaques in AD patients [9,10]. Either an overall increase in Aβ production or an increase in the Aβ42/Aβ40 ratio is correlated with disease progression [11]. 

Cerebral amyloid angiopathy (CAA) is also characterized by amyloid formation of secreted Aβ peptides. In CAA, amyloid deposition occurs on the cerebral blood vessels rather than in the brain parenchyma as in AD. The vascular amyloid results in loss of vessel wall integrity, which leads to vessel hemorrhaging [12,13,14,15]. In contrast to AD, the Aβ40 peptide is the predominant form of Aβ in CAA [16,17]. 

In both AD and CAA, there are familial mutations in APP that enhance disease progression. Understanding how these mutations change the structure or dynamics of APP or the Aβ peptides themselves can shed light on the mechanisms for Aβ toxicity, clearance and amyloid deposition in these two diseases. In AD, there are two major clusters of familial mutations that lead to early onset AD. The first cluster is at the β-secretase cleavage site where mutations can enhance cleavage, leading to an increase in total secreted Aβ [18]. The second cluster is C-terminal to the γ-secretase cleavage site, where mutations result in an increase in Aβ42 relative to Aβ40 [19,20,21]. These sets of familial AD mutants generally result in an increase in the total Aβ produced or in an increase in the Aβ42/Aβ40 ratio, rather than influencing the kinetics of Aβ fibril formation or the structure of the Aβ oligomers or fibrils. 

There are several mutations at positions 22 and 23 in the middle of the Aβ sequence that enhance vascular amyloid deposition. Aβ40-Iowa (D23N) is unique in that no additional familial mutations have been reported at position 23. In CAA cases, the Aβ40-Iowa peptide preferentially deposits on microvessels and capillaries and exhibits a robust perivascular neuroinflammatory response [22]. In addition, this mutation can lead to the formation of fibrils with anti-parallel β-sheet structure in solution under low temperature and low salt conditions [23]. The fibril structures of Aβ40-WT and Aβ40-Iowa both exhibit a bend near position 23 that allows the hydrophobic N-terminal Leu17-Ala21 sequence to pack on the C-terminal Ile31-Val36 sequence. In mature fibrils of the Aβ40-WT peptide with parallel β-sheet structure, the Asp23 side chain forms a salt bridge with Lys28 within the hydrophobic fibril core [24], while in Aβ40-Iowa fibrils with anti-parallel β-sheet structure, Asn23 forms hydrogen-bonds within the fibril core, but Lys28 remains oriented outward toward solvent where it interacts electrostatically with the C-terminal carboxyl group of the neighboring peptide in the fibril [23]. 

Position 22, adjacent to the site of the Iowa mutation, is a second site where mutations occur that enhance CAA. Several mutations have been described at this position, including the Arctic (E22G), Italian (E22K) and Dutch (E22Q) variants. Like Aβ40-Iowa, the E22K and E22G mutations dramatically enhance the rate of fibril formation [25,26]. The E22Q Dutch mutation, on the other hand, exhibits fibrillization kinetics that are only slightly faster than Aβ40-WT [25,26]. This mutation is one of the first CAA mutations to be identified [27]. Both Aβ40-WT and Aβ40-Dutch preferentially deposit on cerebral arterioles and small arteries [27], which contrasts with the location of vascular deposition for Aβ40-Iowa. 

In this study, we address the mechanism(s) of fibril formation of the Aβ40-Dutch and Aβ40-Iowa peptides using spectroscopic methods to follow structural changes that occur in the transition from monomers to fibrils in solution. The pathways for forming these Aβ fibrils are of interest for several reasons. First, familial mutations at positions 22 and 23 of the Aβ sequence lead to faster Aβ aggregation and are associated with CAA [26]. In general, the rate limiting steps in fibril formation for wild-type (WT) Aβ and the familial Aβ mutants are still not well understood. Second, the Aβ40-Dutch and Aβ40-Iowa peptides are considerably more toxic to cultured cerebral vascular smooth muscle cells than the Aβ40-WT peptide [28]. The double mutant Aβ40-Dutch, Iowa (Aβ40-DI) combines the Dutch and Iowa mutations in a single peptide. While this mutant is not found clinically, it serves as a point of comparison for our current studies as it forms fibrils much more rapidly and exhibits a two-fold increase in toxicity relative to either Aβ40-Dutch or Aβ40-Iowa [28]. Finally, soluble oligomers are generally thought to be the toxic species in AD. However, in CAA the disruption of vascular membranes may involve the formation of fibrils rather than oligomers [29,30]. One open question is whether these peptides form well-defined soluble oligomers and protofibrils, similar to Aβ40-WT and Aβ42-WT. 

The time course from monomers to fibrils is followed using FTIR, thioflavin T fluorescence and circular dichroism (CD) spectroscopy. One of the main aims of this study was to distinguish the formation of anti-parallel β-hairpin structure in oligomers and protofibrils from anti-parallel cross-β-sheet structure in mature fibrils. Anti-parallel β-structure resulting from β-hairpin formation in soluble oligomers is often attributed to the presence of cross-β-sheet fibril structure within these oligomers. To reduce the formation of anti-parallel cross-β-sheet structure, the time course measurements were undertaken at 37 °C and with strong agitation, both of which favor the conversion to parallel, in-register fibrils. Our FTIR and thioflavin T fluorescence measurements reveal that the CAA mutant peptides rapidly form a transient anti-parallel β-hairpin intermediate, which has also been reported for the Aβ40-WT [31] and Aβ42-WT peptides [32]. This intermediate occurs when oligomers and protofibrils are observed by atomic force microscopy (AFM) at early time points in the fibrillization process, and when appreciable β-sheet structure has not yet formed as monitored by CD spectroscopy. That is, the anti-parallel β-hairpin structure forms prior to the observation of β-sheet-containing fibrils. β-hairpins are stabilized by intramolecular hydrogen bonding and consequently differ markedly from the anti-parallel β-sheet structure observed for Aβ40-Iowa fibrils [23] or from fibrils formed using Aβ40-DI seeds obtained from transgenic rats [33]. We discuss these results in terms of the pathway(s) for fibril formation, clearance, vascular amyloid formation and cellular toxicity of the Aβ40-Dutch and Aβ40-Iowa mutants in the aging brain. 

## 2. Results

### 2.1. Monomer to Fibril Conversion of Aβ40-WT 

**Figure 1:**
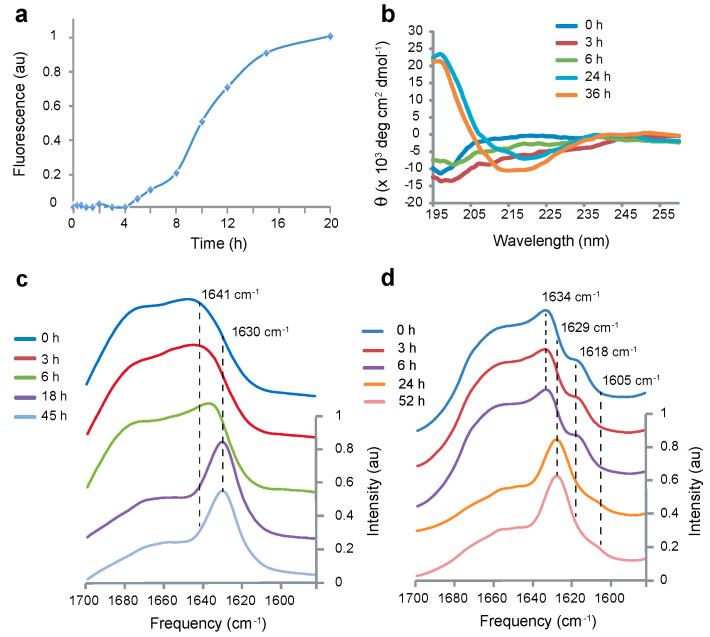
Fibril formation of Aβ40-WT involves a transient anti-parallel non-fibrillar intermediate. Time courses of fibril formation from monomeric Aβ40-WT at 37 °C are shown using thioflavin T fluorescence (**a**), circular dichroism (**b**) and FTIR (**c**,**d**) spectroscopy. Fibril formation monitored by thioflavin T fluorescence at 490 nm increases rapidly after ~8 h of incubation of monomeric Aβ40-WT. The peptide is largely unstructured before the increase in thioflavin T fluorescence. This is observed by both the lack of a CD signal and by the broad amide I band in the FTIR spectrum. Isotopic labeling of the Aβ40-WT peptide with 1-^13^C glycine at Gly33 in (**d**) results in an isotropically shifted peak at ~1618 cm^−1^ that is characteristic of anti-parallel β-structure. We interpret the appearance and disappearance of this band as the conversion of monomeric Aβ40-WT into an anti-parallel β-hairpin that then converts into parallel, in-register β-sheet as fibrils form.

An increase of thioflavin T fluorescence at 490 nm is often used to characterize the kinetics of fibril formation of the Aβ peptides. The time course for fibril formation depends on several key experimental parameters including temperature, concentration and agitation [32]. The temperature (37 °C) and agitation (200 rpm shaking) conditions were chosen to facilitate the transition to fibrils having β-strands associated in a parallel orientation. Aβ40-WT typically has a long lag phase prior to fibril formation compared to Aβ42-WT or to Aβ40 with mutations at positions 22 and 23. Under the conditions of our experiments (50 mM NaCl, 10 mM phosphate buffer, pH 7.2, 37 °C, 100 μM Aβ), the lag phase observed in fluorescence experiments is ~8 h (Figure 1a). In contrast, under quiescent conditions at lower temperatures, the lag phase in Aβ40-WT fibrillization is several days. The relatively short lag phase observed here is largely attributed to strong agitation of the sample. In addition, strong agitation facilitates the conversion of the Aβ40-Iowa peptide to a parallel, in-register fibril structure [34]. The samples were diluted prior to measurements in order to shift the equilibrium between monomers, oligomers and protofibrils toward monomer. Under these conditions, the fluorescence readings are more reflective of stable fibrils [32]. 

CD and FTIR spectroscopy are both sensitive to protein secondary structure. Monomers of the Aβ40-WT peptides formed at low temperature (4 °C) adopt largely random coil structure, which exhibits a negative band at ~200 nm in CD spectra. The negative ellipticity of the CD spectrum at *t* = 0 h is not strong, suggesting the presence of β-structure already at this time point (the absolute value of the negative molar ellipticity of random coil is roughly equal to the positive molar ellipticity of β-sheet at 200 nm [35,36]). An increase in temperature enhances the association of Aβ monomers into fibrils with β-sheet structure, seen as a positive CD absorption band between 190 and 200 nm and a broad negative CD band between 210 and 220 nm. Using these structural markers, β-sheet forms in the Aβ40-WT peptide between 6 and 24 h under the conditions used (Figure 1b). These changes correlate with the increase in thioflavin T fluorescence after 8 h in Figure 1a. 

We have previously shown using FTIR spectroscopy that the Aβ42-WT peptide forms a transient β-hairpin prior to association into β-sheet fibrils [32]. The β-hairpin is formed by intramolecular hydrogen bonding of two anti-parallel β-strands in the Aβ peptide. The β-strands roughly correspond to the two hydrophobic stretches in the sequence (Leu17-Ala21 and Ile31-Val36). As with fluorescence and CD spectroscopy, the time course of fibril formation can be followed by FTIR spectroscopy. The Aβ40 monomer exhibits a broad band between 1640 and 1680 cm^−1^, characteristic of random coil. The featureless amide I band in the FTIR spectrum at *t* = 0 h for the unlabeled peptide is characteristic of random coil (e.g., it spans the amide I range of vibrational frequencies and exhibits no defined, sharp features). The formation of β-sheet results in a sharp amide I IR band at ~1630 cm^−1^. The FTIR time series (Figure 1c) shows the transition to β-sheet in the same time frame as the CD and fluorescence measurements.

Distinguishing parallel and anti-parallel β-sheets is generally a challenge when using FTIR. A weak band at 1695 cm^−1^ is often used as a diagnostic for anti-parallel β-sheet structure [37]. However, this band is often obscured by the broad random coil background at early time points on the pathway to fibrils. For Aβ42-WT, our approach has been to isotopically label the backbone carbonyl with ^13^C at Gly33 within the C-terminal β-strand of the peptide [32], and for C99 we have incorporated ^13^C labels in the Leu17-Ala21 sequence in the middle of the peptide sequence [38]. The ^13^C=O label results in a splitting of the amide I normal mode for anti-parallel β-sheet with an increase in the intensity of a low frequency band at ~1617 cm^−1^ [39,40]. In parallel β-sheet with this labeling scheme, the low frequency band shifts to ~1603 cm^−1^ and loses intensity [32]. Figure 1d presents an FTIR time series using 1-^13^C Gly33 labeled Aβ40-WT. An isotope-shifted resonance at 1618 cm^−1^ is observed between 0 and 6 h. This resonance shifts to 1605 cm^−1^ and loses intensity as fibrils form. These changes are similar to those observed for Aβ42-WT and are attributed to a transition from an intermediate with anti-parallel β-hairpin structure to fibrils with parallel cross-β-sheet structure [32]. 

### 2.2. Aβ40-Dutch and Aβ40-Iowa form Transient Anti-Parallel β-Hairpins 

**Figure 2:**
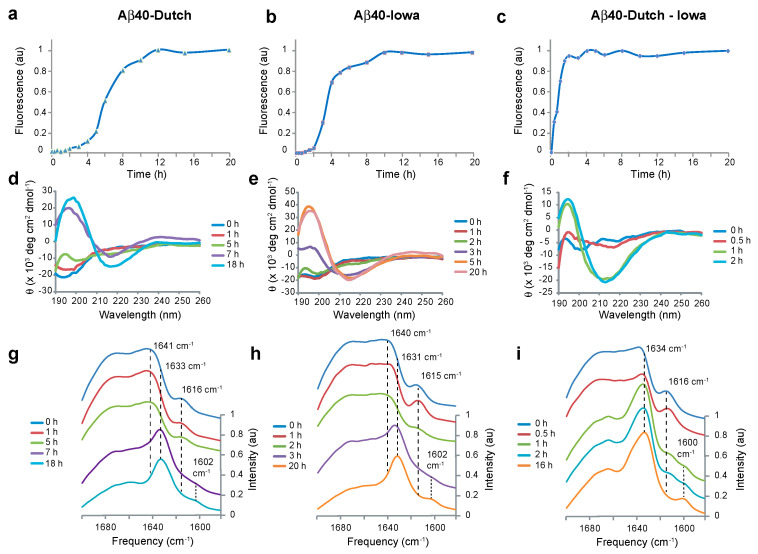
Fibril formation of Aβ40-Iowa, Dutch and Dutch-Iowa mutants involves a transient anti-parallel non-fibrillar intermediate. Time courses of fibril formation from monomeric Aβ40 peptides at 37 °C are shown using thioflavin T fluorescence (**a**–**c**), circular dichroism (**d**–**f**) and FTIR (**g**–**i**) spectroscopy. The time courses for the mutant peptide for fibril formation monitored by thioflavin T fluorescence at 490 nm is much more rapid than for Aβ40-WT. However, for each peptide when labeled with 1-^13^C Gly33, there is observation of a β-hairpin intermediate as fibrils form.

Several studies have previously shown that mutations at positions 22 and 23 of Aβ40 result in fibrils forming much more rapidly than Aβ40-WT [26,28]. On the basis of thioflavin T fluorescence (Figure 2a–c), Aβ40-Dutch fibrils form within 8 h at 37 °C, while Aβ40-Iowa fibrils form within 5 to 6 h under the same conditions as used for Aβ40-WT in Figure 1. The combination of the Dutch and Iowa mutations in a single peptide has a markedly stronger phenotype than either of the single mutations [41]. The expression of the double mutant Aβ40-DI peptide in transgenic mice decreases the age of onset of vascular amyloid deposition and increases the amount of vascular amyloid formed relative to either Aβ40-Iowa or Aβ40-Dutch [41]. In solution, the Aβ40-DI double mutant exhibits more rapid fibril formation using thioflavin T than the single mutants (Figure 2c). 

The time scale for fibril formation for Aβ40-Dutch, Aβ40-Iowa and Aβ40-DI is also reflected in the time scale for β-sheet formation by CD spectroscopy (Figure 2d–f). As observed in the CD spectrum of Aβ40-WT, the *t* = 0 h spectra of the familial mutants of Aβ40 do not exhibit an intense random coil band at 200 nm, suggesting the presence of β-structure already in these samples, the positive ellipticity of which cancels the negative ellipticity characteristic of random coil. The absence of a negative random coil band is most apparent for the Aβ40-DI peptide, which exhibits the fastest fibrillization rate by thioflavin T fluorescence. 

Using the same 1-^13^C Gly33 labeling scheme as used in Figure 1d, we address whether the Aβ40 mutants exhibit a similar anti-parallel intermediate as in Aβ40-WT. The FTIR time series of Aβ40-Dutch, Aβ40-Iowa and Aβ40-DI reveal the transient appearance and disappearance of an isotope shifted band at ~1615 cm^−1^ prior to fibril formation (Figure 2g–i). For all peptides, the isotope shifted band is observed at *t* = 0 h (i.e., immediately after forming the monomers at 4 °C and layering the sample on the ATR plate at room temperature for FTIR measurements). The intensity of the 1615 cm^−1^ band increases slightly for the Aβ40-Iowa and Aβ40-DI peptides before shifting and losing intensity. For Aβ40-DI, the presence of both the 1600 and 1617 cm^−1^ bands were observed in the spectra obtained at 1 and 2 h prior to a shift in the low frequency band at 1600 cm^−1^ at longer times.

**Figure 3:**
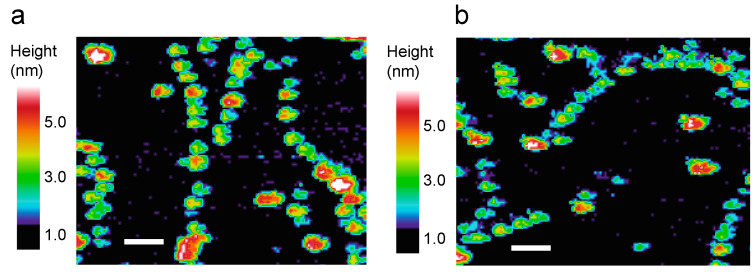
Single touch AFM images of Aβ40-Dutch (**a**) and Aβ40-Iowa (**b**). Images were obtained after 30 min of incubation at room temperature. Heights of the aggregates above the mica surface (in nm) are color-coded. Scale bars = 50 nm.

The spectroscopic data above define a window prior to fibril formation where there is a transient appearance of intensity in the isotope-shifted IR band attributed to β-hairpin secondary structure. In Figure 3, single touch AFM images are presented after 30 min of incubation of monomeric Aβ40-Dutch and Aβ40-Iowa. The images capture the formation of low molecular weight oligomers with heights of 2–3 nm that are associating to form protofibrils.

Higher molecular weight oligomers with heights of 5–7 nm are also observed, but are much less abundant and do not increase with time as observed in the pathway to fibrils using the Aβ42-WT peptide [42]. Although not clear from these single images, the Aβ40-Dutch peptide tends to exhibit more aggregated species than Aβ40-Iowa. The fibrils formed from the protofibrils of Aβ40-Iowa typically have heights of ~3 nm [34], similar to those observed for the Aβ40-Arctic mutation, but distinct from the ~6–7 nm heights observed for Aβ40-WT [43] and from the high molecular weight oligomers in Aβ42-WT [42].

## 3. Discussion

Our current results show that the Aβ40-Iowa and Aβ40-Dutch peptides form a transient β-hairpin intermediate, a feature shared by wild-type Aβ40 and Aβ42. Evidence for an anti-parallel β-hairpin forming in Aβ40-WT, Aβ40-Dutch and Aβ40-Iowa comes from FTIR measurements. In the FTIR spectra, the signature anti-parallel β-strand peak appears immediately after the Aβ40-WT peptide is filtered and layered on the ATR plate for FTIR measurements. Nevertheless, there is substantial random coil in these peptides. Solution NMR studies on Aβ40-WT indicate that the β-hairpin structure is in chemical exchange with random coil with the equilibrium in favor of the random coil structure [44]. We interpret the broad band between 1660 and 1690 cm^−1^ as random coil. The ~1634 and 1641 cm^−1^ bands observed for ^13^C-labeled and unlabeled Aβ40-WT are characteristic of nascent β-strands. Mature fibrils with well-formed β-sheet exhibit a sharp amide I band from 1626 to 1630 cm^−1^ in unlabeled fibrils. The split bands at 1618 and 1634 cm^−1^ in the peptide containing a ^13^C=O label at Gly33 are interpreted as arising from anti-parallel β-strands. We did not observe a defined peak at 1672 cm^−1^ characteristic of α-sheet structure [45].

The proposed β-hairpin structure associated with this transient intermediate state has intra-molecular hydrogen bonding between a β-strand centered on the hydrophobic Leu17-Ala21 and Ile31-Gly37 sequences. The model that emerges from these and other studies [31,32] is that the first step in conversion of monomeric Aβ into fibrils is the rapid conversion to a β-hairpin structure stabilized by intramolecular hydrogen bonding. This mechanism is likely to be concentration independent since it is an intramolecular interaction. As a result, the formation should occur at low physiological concentrations. Recent structural studies of C99 show that sequence from Tyr10 to Ala21 forms a β-hairpin that associates with the membrane bilayer [46]. As a result, the Leu17-Ala21 β-strand is primed to form a β-hairpin with the C-terminal Ile31-Gly37 sequence following γ-secretase cleavage of C99 and release of the Aβ peptide from the enzyme [46].

There is a wide range of data supporting the transient formation of a β-hairpin intermediate on the pathway to Aβ fibrils. One line of evidence comes from protein engineering studies in which β-hairpins are stabilized by an intra-molecular disulfide bond [47,48,49]. The CC crosslink prevents intermolecular hydrogen bonding in the cross-β-sheet structures characteristic of mature fibrils. Aβ40-CC and Aβ42-CC both spontaneously form stable oligomers and protofibrils, but both are unable to convert into amyloid fibrils. Conformation-specific antibodies used to detect Aβ aggregates in vivo indicate that the wild-type oligomer structure is preserved and stabilized in the Aβ-CC oligomers.

In a similar fashion, Tycko and Meredith [50] synthesized Aβ40-lactam(D23/K28), which contains a lactam bridge between the side chains of Asp23 and Lys28. Rather than locking the monomer in the β-hairpin conformation, the lactam locks the monomer in the conformation the Aβ40 peptide adopts in cross-β-sheet fibrils. Aβ40-lactam (D23/K28) forms fibrils similar to those formed by Aβ40-WT. However, fibril formation occurs without a lag phase and at a rate ~1000-fold greater than for Aβ40-WT.

Since the formation of anti-parallel β hairpin occurs rapidly, the rate-determining step in fibril formation likely involves the rotation of the individual β-strands to form intermolecular hydrogen bonds in cross-β-sheet fibrils. For Aβ40-WT, this involves the rotation of the negatively charged Asp23 and positively charged Lys28 into the hydrophobic interior of the nascent fibril where they are stabilized by salt-bridge formation. Separately, rotation of either the charged Asp23 or Lys28 side chains into the hydrophobic fibril core is energetically unfavorable, which explains the rapid fibril kinetics and lack of a lag phase when these residues are cross-linked in the Aβ40-lactam (D23/K28).

In the Iowa mutant (D23N), the uncharged Asn side chain does not have this energetic barrier to rotation. In the anti-parallel fibril structure of Aβ40-Iowa, Asn23 is hydrogen bonded with Gln15 and Asn27, whereas Lys28 is oriented outward where it interacts with the C-terminus of the neighboring peptide in anti-parallel fibrils [23]. The outward orientation of Lys28 is also seen in Aβ42-WT where it forms a salt bridge with the negative charge on the C-terminal carboxyl group [51,52].

Unlike Aβ40-Iowa, a fibril structure has not been determined for Aβ40-Dutch. Solid-state NMR studies on both the E22G [53] and E22K [54] mutants suggest polymorphism in their fibril structure. For E22K, NMR experiments have shown that the structural polymorphism reflects one population where Asp23 rotates inward to form a salt bridge with Lys28 and a second population where the Asp23 side chain remains oriented outward and interacts electrostatically with the Lys22 side chain [54], indicating that there is at least one fibril population with a structure that is similar to Aβ40-WT.

Although all of the peptides studied here form a transient β-hairpin intermediate with anti-parallel β-strands, the kinetics of fibril formation are quite different. Using AFM, we showed that at 100 μM Aβ concentration both Aβ40-Iowa and Aβ40-Dutch form oligomers that predominantly have heights of 2–3 nm. These oligomers laterally associate and merge to form cross β-sheet fibrils in a fashion previously described for Aβ42-WT at high Aβ concentrations [32]. Unlike Aβ42-WT, the heights of the initial fibrils formed are lower. The oligomeric intermediates indicate that the fibrils form via nucleated conformation conversion. However, the lower heights observed for the fibrils are consistent with nucleated polymerization pathways that involve monomer addition to nucleation sites and occur at lower Aβ concentrations [32,55]. Fibrils with low heights are observed for Aβ42-WT at low Aβ concentrations where there is appreciable Aβ monomers in solution [56]. In addition, the E22Q and D23N mutations occur close to or within the turn region in the β-hairpin and likely control the final β-hairpin structure and resulting fibrils [57].

The E22Q and D23N mutations are adjacent to one another in the Aβ40 sequence and both result from substitution of a negatively charged residue to a neutral amine. One possible contributor to enhanced deposition of the familial CAA mutant peptides involves the mechanism for the clearance of the Aβ peptides from the brain [58,59]. In this regard, there are a number of different pathways for clearance including degradation by enzymes such as neprilysin, uptake and degradation by astrocytes and microglia, transport out of the interstitial fluid through the cerebral spinal fluid and efflux across the blood–brain barrier into the blood stream. The structure (monomer, oligomer, fibril) of the Aβ peptide likely determines the pathway(s) taken. Since Aβ40-WT, Aβ40-Dutch and Aβ40-Iowa adopt a similar β-hairpin structure to the monomer, it is likely the differences in the clearance pathway(s) depend on the rate of fibril formation and stability of the protofibrils or fibrils during the aggregation process. Stable fibrils are less likely to cross membrane barriers that would be required for transport out of the interstitial brain fluid via the cerebral spinal fluid or blood stream.

## 4. Materials and Methods

### 4.1. Sample Preparation

Aβ peptides were synthesized using N-t-Boc-chemistry (ERI-Amyloid, Waterbury, CT, USA) and purified by high-performance liquid chromatography. The mass of the purified peptide was measured using matrix-assisted laser desorption or electrospray ionization mass spectrometry, and was consistent with the calculated mass for the peptide. On the basis of analytical reverse phase high-performance liquid chromatography and mass spectrometry, the purity of the peptides was generally >98%.

Purified, lyophilized Aβ40 peptides (ERI Amyloid Laboratory, Oxford, CT, USA) were dissolved in 100 mM NaOH at a concentration of 2.2 mM, then diluted in buffer (10 mM phosphate, 50 mM NaCl) at low temperature (4 °C) and titrated to pH 7.4. The Aβ solutions were then filtered two times with 0.2-micron cellulose acetate filters to remove insoluble aggregates that can nucleate and influence aggregation. The Aβ concentrations were determined by the absorption at 270 nm using a molar extinction coefficient of ε = 1405 cm^−1^·M^−1^. To initiate Aβ aggregation, the solutions of monomeric peptide at 4 °C were placed in a 37 °C incubator and shaken at 200 rpm. For AFM, FTIR and fluorescence measurements, aliquots of the peptide solution were removed at time points between 0 and 50 h.

### 4.2. FTIR Spectroscopy

FTIR spectroscopy was performed with a Bruker IFS 66V/S spectrometer with a liquid nitrogen cooled mercury–cadmium–telluride detector. Spectra were recorded with a spectral resolution of 4 cm^−1^. The internal reflection element was a germanium ATR plate. Samples were prepared by spreading 50–100 μL of 100 μM in 10 mM phosphate and 50 mM NaCl buffer on the plate surface and by drying under N_2_ or air. One thousand scans were averaged for each spectrum. For these experiments, the samples were incubated at 37 °C with 200 rpm agitation and then removed for the FTIR time points. The FTIR measurements were carried out at room temperature.

### 4.3. Circular Dichroism (CD) Spectroscopy

CD spectra were obtained on an Olis RSM CD spectrophotometer (Olis Inc., Bogart, GA, USA) using a 1 mm width quartz cuvette at an Aβ concentration of 100 μM in 10 mM phosphate and 50 mM NaCl buffer. The temperature was maintained at 37 °C.

### 4.4. Thioflavin T Fluorescence Spectroscopy

Fluorescence experiments were performed using a Horiba Jobin Yvon Fluorolog FL3-22 spectrofluorometer. For these experiments, the samples were incubated at 37 °C with 200 rpm agitation as in the CD and FTIR measurements and then removed for the thioflavin T fluorescence time points. At each time point, aliquots were taken and mixed with 30 μM thioflavin T to produce mixtures with peptide/thioflavin T ratio β of 1:20. Thioflavin T fluorescence emission spectra were obtained from 475 to 550 nm using an excitation wavelength of 461 nm.

### 4.5. Atomic Force Microscopy

Single touch AFM images were obtained using a MultiMode microscope (Digital Instruments, Santa Barbara, CA, USA) with a custom-built controller (LifeAFM, Port Jefferson, NY, USA) that allows one low force contact of the AFM tip to the sample surface per pixel [56]. Super-sharp silicon probes with a tip width of 3–5 nm (at a height of 2 nm) were modified for magnetic retraction by attachment of samarium cobalt particles. Samples for AFM were diluted with MilliQ water from a 100 μM Aβ concentration in 10 mM phosphate and 50 mM NaCl buffer to a concentration of 0.5 μM Aβ and then deposited onto freshly cleaved ruby mica (S & J Trading, Glen Oaks, NY, USA) and imaged under hydrated conditions.

## Data Availability

The data presented in this study are contained within the article.

## Abbreviations

AFM	Atomic force microscopy
Aβ	Amyloid β
AD	Alzheimer’s disease
APP	Amyloid precursor protein
CAA	Cerebral amyloid angiopathy
CTF	C-terminal fragment
FTIR	Fourier-transformed infrared
TM	Transmembrane
WT	Wild-type
